# Qualitative and Quantitative Wall Enhancement Analyses in Unruptured Aneurysms Are Associated With an Increased Risk of Aneurysm Instability

**DOI:** 10.3389/fnins.2020.580205

**Published:** 2020-12-10

**Authors:** Yi Zhang, Qichang Fu, Yuting Wang, Jingliang Cheng, Cuiping Ren, Sheng Guan, Chengcheng Zhu

**Affiliations:** ^1^Department of Magnetic Resonance, The First Affiliated Hospital of Zhengzhou University, Zhengzhou, China; ^2^Department of Radiology, Sichuan Provincial People’s Hospital, University of Electronic Science and Technology of China, Chengdu, China; ^3^Department of Interventional neuroradiology, The First Affiliated Hospital of Zhengzhou University, Zhengzhou, China; ^4^Department of Radiology, University of Washington, Seattle, WA, United States; ^5^Department of Radiology and Biomedical Imaging, University of California, San Francisco, CA, United States

**Keywords:** ELAPSS and PHASES scores, intracranial aneurysms, quantitative analysis, vessel wall MRI, aneurysm wall enhancement

## Abstract

**Objective:**

Intracranial aneurysm wall enhancement (AWE) is independently associated with unstable aneurysms. However, a quantitative analysis of wall enhancement is lacking. This study aims to investigate the relationship between qualitative and quantitative wall enhancement indices (WEIs), traditional risk factors for aneurysms, and clinical ELAPSS/PHASES scores in a large cohort of intracranial saccular aneurysms.

**Materials and Methods:**

In this cross-sectional study, a total of 174 patients (mean age 60.4 ± 9.5 years; 53% women) with 248 asymptomatic unruptured intracranial aneurysms underwent pre- and post-contrast black-blood magnetic resonance imaging (MRI). The extent of AWE was defined as non-AWE (pattern 0), focal AWE (pattern 1), or circumferential AWE (pattern 2). WEI was calculated using wall signal intensities on pre- and post-contrast images. Predicted 3- and 5-year growth risk and 5-year rupture risk were obtained from ELAPSS and PHASES scores, respectively. Uni- and multivariate analyses were conducted to explore the relationship between AWE characteristics, risk-related factors, and aneurysm instability.

**Results:**

Aneurysm size [odds ratio (OR), 1.3; 95% confidence interval (CI), 1.2–1.4; *P* < 0.001], non-internal carotid artery/middle cerebral artery location (OR, 1.9; 95% CI, 1.0–3.6; *P* = 0.045), and irregular shape (OR, 2.4; 95% CI, 1.2–4.5; *P* = 0.009) were independently associated with AWE. For aneurysms with AWE, the estimated 3- and 5-year growth risk (25.3 ± 13.0% and 38.0 ± 17.4%) and the 5-year rupture risk (3.9 ± 5.2%) were 1.9–3.3 times higher than those for aneurysms without AWE (12.8 ± 9.1%, 20.3 ± 13.0%, and 1.2 ± 1.6%, respectively; all *P* < 0.001). Larger areas and higher WEIs of enhancement positively correlated with aneurysm size (*r* = 0.43 and 0.38, respectively), 3- and 5-year growth risk, and 5-year rupture risk (*r* = 0.49 and 0.40, *r* = 0.49 and 0.40, *r* = 0.36 and 0.24, respectively; all *P* < 0.001). In sum, a larger aneurysm size, non-internal carotid artery/middle cerebral artery location, and irregular shape were independently associated with AWE. Larger areas and higher WEIs were associated with an increased risk of aneurysm growth and rupture. These findings suggest that quantitative AWE metrics should be considered in future large-scale longitudinal studies to evaluate their value in aneurysm risk management.

## Introduction

The prevalence of intracranial aneurysms is approximately 3% (Vlak et al., 2011). The growing availability of conventional computed tomography angiography and magnetic resonance angiography in clinical practice has led to increased detection of unruptured incidental aneurysms. The annual rupture rate of intracranial aneurysms is approximately 1.2% (Juvela, 2018). Aneurysmal subarachnoid hemorrhage (SAH) is a risk for aneurysm rupture. Perturbations in cerebrospinal fluid circulation and the presence of intraventricular hemorrhage may lead to impairments in daily life (Hütter et al., 2001). The incidence of treatment-related complications is significant (Brunozzi et al., 2019). Therefore, for unruptured intracranial aneurysms (UIAs), individual prediction of risk for instability (growth and rupture) is required to guide clinical decision making and patient management.

ELAPSS (Backes et al., 2017) and PHASES (Greving et al., 2014) scores are based on large-scale multi-center studies and are popular clinical tools for the evaluation of aneurysm growth and rupture. Previous studies have reported that aneurysm wall enhancement (AWE) identified by black-blood MRI was associated with aneurysm rupture (Matouk et al., 2013; Nagahata et al., 2016). Several studies have investigated the relationship between AWE and conventional risk factors, including aneurysm size and location. Zhu et al. (2018) reported that stronger signal intensity and larger areas of AWE were correlated with aneurysm size, size ratio, and estimated rupture risk based on UCAS (UCAS Japan Investigators et al., 2012) and PHASES (Greving et al., 2014) calculators. However, most previous studies used qualitative grading of AWE by radiologists. In this regard, the quantification of AWE using objective methods is lacking to date. Our study therefore aimed to investigate the relationship between qualitative and quantitative wall enhancement indices (WEIs), traditional risk factors for aneurysms and clinical ELAPSS/PHASES scores in a large cross-sectional cohort of intracranial saccular aneurysms.

## Materials and Methods

The ethics committee of the First Affiliated Hospital of Zhengzhou University approved this prospective, cross-sectional study. Written informed consent was obtained from all patients.

### Clinical Materials

Patients with intracranial aneurysms attending our hospital from October 2014 to October 2019 were recruited for this cross-sectional study. All patients underwent both three-dimensional (3D) digital subtraction angiography and 3.0 T high-resolution MRI. Exclusion criteria were (1) ruptured aneurysm, (2) aneurysms without digital angiograph imaging, with unsatisfactory image quality caused by motion artifacts, or with incomplete clinical records, (3) dissecting, fusiform, conus arteriosus or adjacent to cavernous sinus, and (4) symptomatic aneurysms. Possible clinical risk-related factors were exported from the clinical database, including age, sex, early SAH history, hypertension, and current smoking history.

### Imaging Protocol

Magnetic resonance scans were performed on a 3.0-T MR scanner (Verio, Siemens Healthcare, Erlangen, Germany) with a 16-channel head coil in a subset of patients during initial stages of the study. In subsequent stages, MR imaging of patients was performed on a 3.0-T MR scanner (Prisma, Siemens Healthcare, Erlangen, Germany) with a 64-channel head coil.

First, 3D time-of-flight magnetic resonance angiography was used to locate the aneurysm. The scanning parameters were as follows: repetition time (TR)/echo time (TE), 20.0/3.7 ms; layer thickness = 0.6 mm; 200 mm × 80 mm field of view; 176 slices; resolution 0.6 mm with a scan time of 2 min 29 s. Initially, 30 patients with 35 aneurysms underwent T1-weighted 2D black-blood fast-spin-echo imaging. The scanning parameters of pre- and post-contrast 2D T1-weighted images were consistent: TR/TE, 430/10 ms; layer thickness, 2.0 mm; 140 mm × 81 mm field of view; seven to twelve slices; and resolution 0.5 mm. Scan time was 4 min 55 s to 8 min 34s. At a later stage in the study, 144 patients with 213 aneurysms were scanned using T1-weighted 3D fast-spin-echo with variable flip angle trains (aka 3D-SPACE) when available. The scanning parameters of pre- and post-contrast 3D T1-weighted images were consistent: TR/TE 800/14 ms; layer thickness = 0.6 mm; 192 mm × 192 mm field of view; 224 slices; and resolution 0.6 mm. Scan time was 7 min 36 s. Post-contrast 2D/3D T1-weighted images were scanned 5 min after injection of the contrast agent Gd-DTPA (Magnevist; Bayer HealthCare Pharmaceuticals, Germany) at a dose of 0.1 mmol/kg and a flow rate of 1.5 mL/s.

### Qualitative Measurement of Aneurysm Wall Enhancement

The maximum diameter of the aneurysm dome was defined as the aneurysm size based on 3D angiographic data by identifying the best viewing angle for morphologic measurement. Irregular aneurysm shape was defined as the presence of a daughter sac or blebs.

**FIGURE 1:**
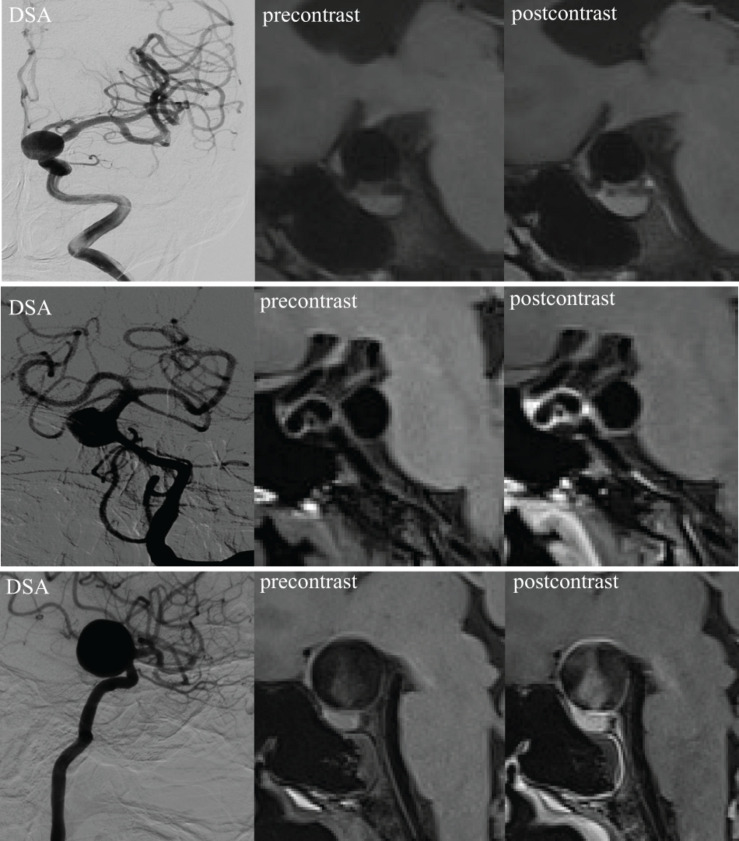
3D high-resolution MRI demonstrating an aneurysm wall without enhancement (pattern 0, first row), focal enhancement (pattern 1, second row), and circumferential enhancement (pattern 2, third row). DSA, digital subtraction angiography.

Our AWE classification was adopted and adjusted from a previous publication (Edjlali et al., 2018). The results were defined as none (no wall enhancement or similar degree of enhancement to the normal arterial wall; pattern 0, the first row in Figure 1), focal (AWE area involved the dome, the intermediate portion, the neck, or a bleb; pattern 1, the second row in Figure 1), and circumferential enhancement (AWE area involved the entire aneurysm; pattern 2, the third row in Figure 1). The MRI images were analyzed separately by two experienced radiologists (JC and YZ with 30 and 20 years of experience in neurovascular imaging, respectively). Inconsistencies in imaging evaluation were resolved by reaching a consensus after consultation.

### Quantitative Measurement of Aneurysm Wall Enhancement

**FIGURE 2:**
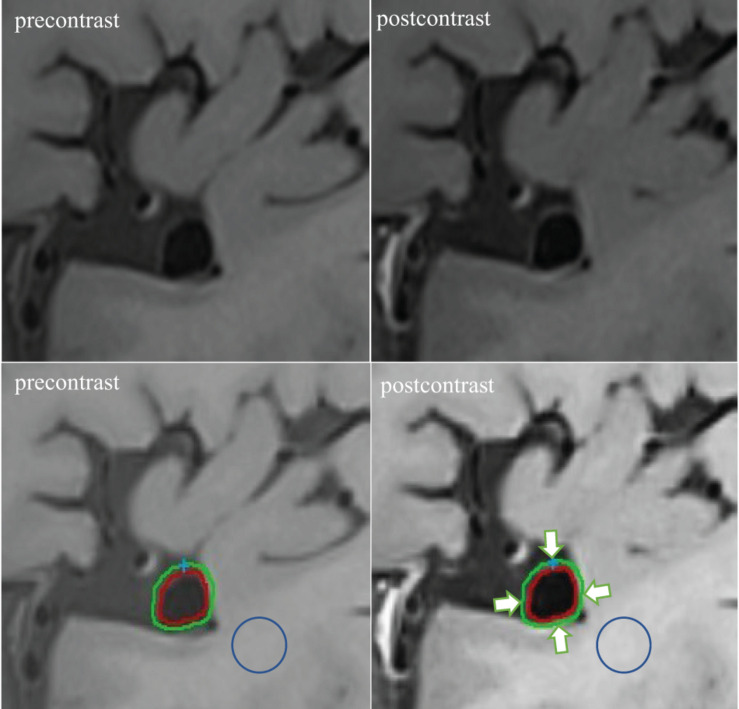
Example of WEI measurement. The post-contrast vessel wall imaging and matched pre-contrast imaging are depicted (upper right and upper left). The software automatically segmented the inner and outer wall contours of the aneurysm in the post-contrast and corresponding pre-contrast imaging (lower right and lower left). Subsequently, all contours in each layer of an aneurysm were automatically segmented into four quarters (arrows indicated). Similar methods were used to measure the average SI of regions of interest on the adjacent white matter on the pre- (circle: lower left) and post-contrast (circle: lower right) vessel wall imaging.

For quantitative analysis of the vessel wall MRI images, two experienced neuroradiologists (QF and YZ with 10 and 5 years of clinical experience in neuroradiology, respectively) who were blinded to the clinical data and digital subtraction angiography results, evaluated the images of UIAs using Vessel-MASS software (MEDIS, Version: 2014-EXP). The software we used had a registration function which was applied to correct for rigid motion during the two scans. Three slices with the most significant enhancement on the post-contrast vessel wall were selected. Images were selected by raters who subsequently traced the lumen and outer boundaries of the aneurysm wall through the optimal display angle (coronal, sagittal, or axial based on the specific geometry of the aneurysm) (Figure 2, lower right). The software automatically matched the slice locations to the corresponding pre-contrast images (Figure 2, lower left). The inner and outer wall contours of the aneurysm were automatically segmented by the software, with manual adjustment of the contours if the neuroradiologist considered the tracings unsatisfactory. Subsequently, all contours in each layer of an aneurysm were automatically segmented into four quarters (arrows in Figure 2, lower right). The mean signal intensity (SI) in each quarter (defined as quarter SI) was automatically calculated. The quarter with the highest mean SI was selected for each slice, and the average value of the three quarters from the corresponding three slices was used to represent the SI of the aneurysm wall. This method allowed capture of the most enhanced part of the aneurysm wall. The average SI of the aneurysm wall at the corresponding position on the pre-contrast vessel wall imaging was obtained using similar methods. To normalize the SI, similar methods were used to measure the average SI of regions of interest on the adjacent white matter in the pre- (Figure 2, lower left) and post-contrast (Figure 2, lower right) vessel wall images. In our cohort, all patients had routine brain MRI including T1-weighted, T2-weighted, and DWI. When drawing the ROI, we had avoided the location of ischemia or hypoperfusion as identified on brain MRI. And in such an asymptomatic patient cohort, there were very few patients having ischemic events. The software automatically calculated the WEI value using a previously proposed formula (Omodaka et al., 2016) as follows: (SIwall/SIbrain on post-contrast imaging - SIwall/SIbrain on matched pre-contrast imaging)/(SIwall/SIbrain on matched pre-contrast imaging).

### Estimated 3- and 5-Year Aneurysm Growth Risk

We calculated the 3- and 5-year growth risk of each aneurysm based on the ELAPSS score (Backes et al., 2017), coded as follows. E = early SAH, score 0–1; L = aneurysm location, score 0–5; A = age, score 0–1; P = population, score 0; S = aneurysm size, score 0–22; S = aneurysm shape, score 0–4. For instance, a 60-year-old female patient with a 9-mm irregular internal carotid artery aneurysm, hypertension, and an earlier SAH history was scored as follows: E (1) + L (0) + A (0) + P (0) + S (13) + S (4) = 18.

### Estimated 5-Year Aneurysm Rupture Risk

We calculated the 5-year rupture risk of each aneurysm based on the PHASES score (Greving et al., 2014), coded as follows. P = population (given that no specific score for the Chinese population is available, we used 0 points for the North American/European population excepting Finland as a representation); H = hypertension, score 0–1; A = age, score 0–1; S = aneurysm size, score 0–10; E = earlier SAH, score 0–1; S = aneurysm site, score 0–4. For instance, a 70-year-old female patient with a 9-mm internal carotid artery aneurysm, hypertension, and an earlier SAH history was scored as follows: P (0) + H (1) + A (1) + S (3) + E (1) + S (0) = 6. The 5-year rupture risk was calculated using the PHASES calculator (www.kockro.com/en/calculator).

### Statistical Analysis

SPSS 21.0 software (SPSS, version 21.0, Chicago, IL, United States) was used for statistical analysis. Variables were expressed as median (interquartile range) and counts (%). Univariate analyses, including the Mann–Whitney *U*-test and the χ^2^ test were used to determine the variables significantly associated with AWE. Multivariate logistic regression analysis was used to determine the risk factors independently associated with AWE. The odds ratio (OR), a 95% confidence interval (CI), and *P-*value were recorded. Spearman’s rank correlation was used to investigate the relationship between AWE characteristics, including qualitative and quantitative assessments, aneurysm size, and predicted aneurysm instability. Interobserver agreement on the AWE pattern was assessed using the Cohen κ coefficient. The intraclass correlation coefficient (ICC) was used to assess the interobserver variability of the WEI calculations. *P* < 0.05 (two-sided) was considered statistically significant.

## Results

### Basic Characteristics of Patients and Aneurysms

**FIGURE 3:**
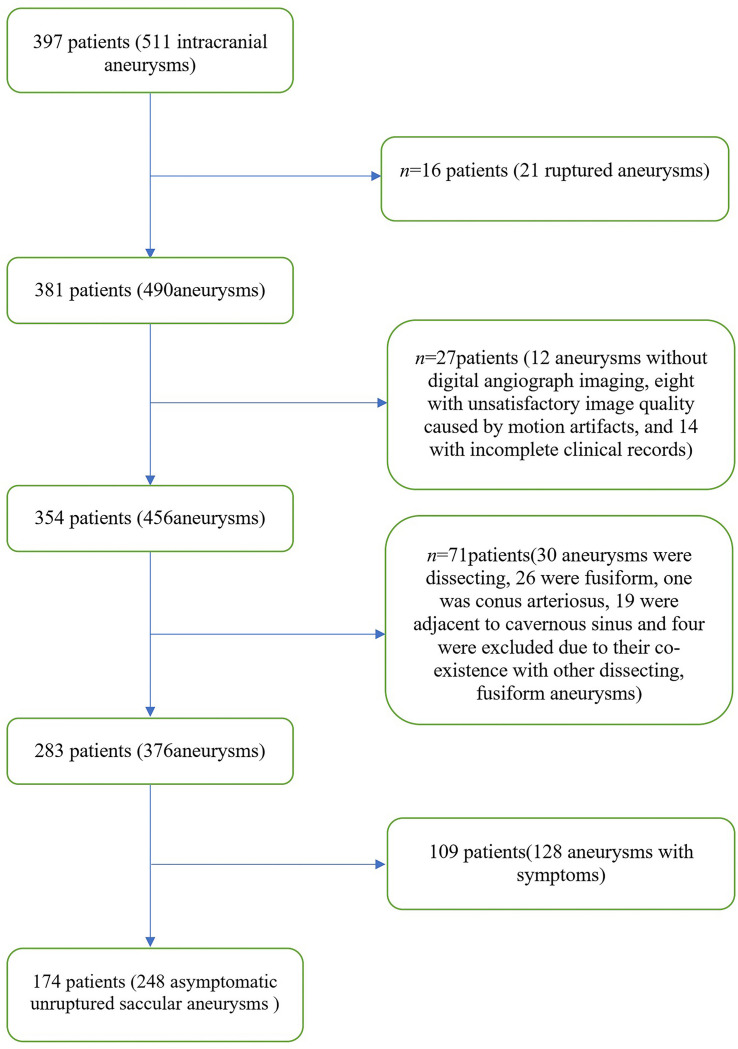
Study flowchart.

A total of 397 patients were included in this study from October 2014 to October 2019. The patient selection flowchart is shown in Figure 3. A final total of 174 patients (mean age, 60.4 ± 9.5 years; 92 women) with 248 asymptomatic unruptured intracranial saccular aneurysms were enrolled. Seven patients (4.0%) had an earlier SAH history, 120 (69.0%) had hypertension, 42 (24.1%) were current smokers, 47 (27.0%) had more than one aneurysms. Patient demographics and aneurysm characteristics are summarized in Table 1.

**TABLE 1:** Characteristics of patients and aneurysms with and without aneurysm wall enhancement.

	Total (*n* = 248)	AWE (*n* = 78)	Non-AWE (*n* = 170)	*P*-value
**Patient information**				
Age	61 (53, 68)	62 (54, 69)	60 (52, 67)	0.088
≥70 years	45 (18.1)	16 (20.5)	29 (17.1)	0.595
Sex (F)	141 (56.9)	43 (55.1)	98 (57.6)	0.783
Early SAH history	10 (4.0)	6 (7.7)	4 (2.4)	0.076
Hypertension	174 (70.2)	59 (75.6)	115 (67.6)	0.233
Current cigarette smoking	56 (22.6)	19 (24.7)	37 (21.6)	0.597
Multiple aneurysms	121 (48.8)	31 (39.7)	91 (52.9)	0.057
**Aneurysm characteristics**				
Size (mm)	4.9 (3.3, 7.0)	6.7 (5.0, 11.5)	4.1 (2.8, 5.7)	<0.001*
1.0–2.9	48 (19.4)	4 (5.1)	44 (25.9)	<0.001*
3.0–4.9	80 (32.3)	12 (15.4)	68 (40)	
5.0–6.9	57 (23.0)	24 (30.8)	33 (19.4)	
7.0–9.9	27 (10.9)	12 (15.4)	15 (8.8)	
≥10	36 (14.5)	26 (33.3)	10 (5.9)	
Location				0.039*
ICA	97 (39.1)	25 (32.1)	72 (42.4)	
MCA	42 (16.9)	11 (14.1)	31 (18.2)	
ACA/AComA	20 (8.1)	3 (3.8)	17 (10)	
PComA/PC	89 (35.9)	39 (50)	50 (29.4)	
Shape (irregular)	77 (31.0)	37 (47.4)	40 (23.5)	<0.001*
WEI	0.3 (0.1, 0.9)	1.2 (1.0, 1.5)	0.2 (0.1, 0.4)	<0.001*
ELAPSS score	12 (7, 19)	19 (15, 27)	10 (5, 16)	<0.001*
0–9	90 (36.3)	6 (7.7)	84 (49.4)	<0.001*
10–20	104 (41.9)	38 (48.7)	66 (38.8)	
≥20	54 (21.8)	34 (43.6)	20 (11.8)	
**Growth risk (%)**				
3-year	16.7 ± 12.0	25.3 ± 13.0	12.8 ± 9.1	<0.001*
5-year	25.9 ± 16.7	38.0 ± 17.4	20.3 ± 13.0	<0.001*
PHASES score	4 (1, 6)	5 (4, 9)	3 (1, 5)	<0.001*
0–4	129 (52.0)	24 (30.8)	105 (61.8)	<0.001*
5–7	78 (31.5)	27 (34.6)	51 (30)	
8–9	19 (7.7)	8 (10.3)	11 (6.5)	
≥10	22 (8.9)	19 (24.4)	3 (1.8)	
**Rupture risk (%)**				
5-year	2.0 ± 3.4	3.9 ± 5.2	1.2 ± 1.6	<0.001*

AWE, aneurysm wall enhancement; SAH, subarachnoid hemorrhage; ICA, internal carotid artery; MCA, middle cerebral artery; ACA, anterior cerebral artery; AComA, anterior communicating artery; PComA, posterior communicating artery; PC, posterior circulation; WEI, wall enhancement index. Variables are expressed as median (interquartile range) and counts (%). *P<0.05.

The median and interquartile range of aneurysmal size were 4.9 mm and 3.3–7.0 mm, respectively. A total of 97 aneurysms (39.1%) were observed in the internal carotid artery (ICA), 42 (16.9%) in the middle cerebral artery (MCA), seven (2.8%) in the anterior cerebral artery (ACA), 13 (5.2%) in the anterior communicating artery (AComA), 39 (15.7%) in the posterior communicating artery (PComA), and 50 (20.2%) aneurysms in the posterior circulation (PC).

### Risk Factors Related to Aneurysm Wall Enhancement

Of the 248 asymptomatic UIAs, 78 aneurysms (31.5%) demonstrated AWE. Among them, 34 exhibited focal AWE (pattern 1) and 44 exhibited circumferential AWE (pattern 2). For the aneurysms with focal enhancement (*n* = 34), most of them (*n* = 21) were at the dome, eight at the lateral wall, and only five were at the base. For the aneurysms with circumferential enhancement, the enhancement presented diffusely at all locations. The interobserver agreement on the AWE patterns and WEI calculations (κ = 0.91; intraclass correlation coefficient = 0.975, respectively) were both excellent.

**FIGURE 4:**
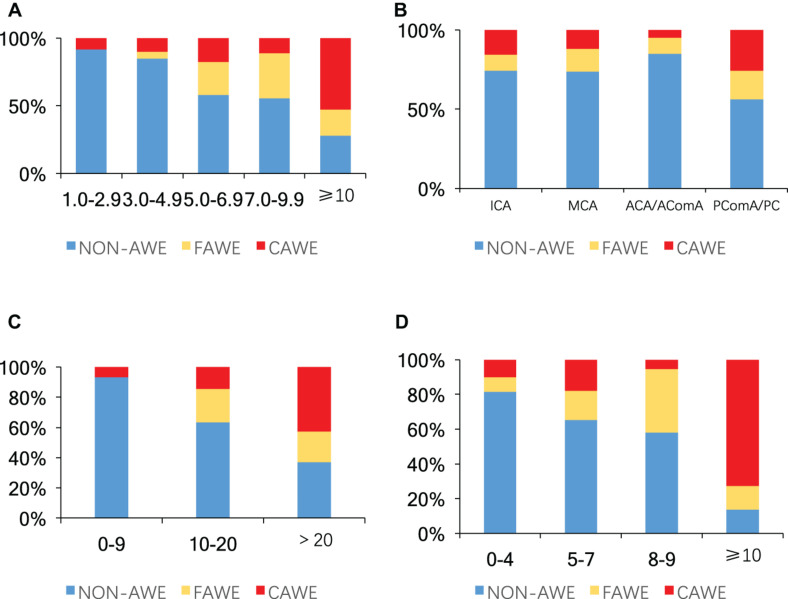
Proportions of aneurysms without enhancement, focal enhancement, and circumferential enhancement according to: **(A)** aneurysm size (mm), **(B)** location, **(C)** ELAPSS score, and **(D)** PHASES score (*P* < 0.001, *P* = 0.013, *P* < 0.001, and *P* < 0.001, respectively). Blue, non-aneurysm wall enhancement; yellow, focal aneurysm wall enhancement (FAWE); red, circumferential aneurysm wall enhancement (CAWE); AWE, aneurysm wall enhancement; ICA, internal carotid artery; MCA, middle cerebral artery; ACA, anterior cerebral artery; AComA, anterior communicating artery; PComA, posterior communicating artery; PC, posterior circulation.

**FIGURE 5:**
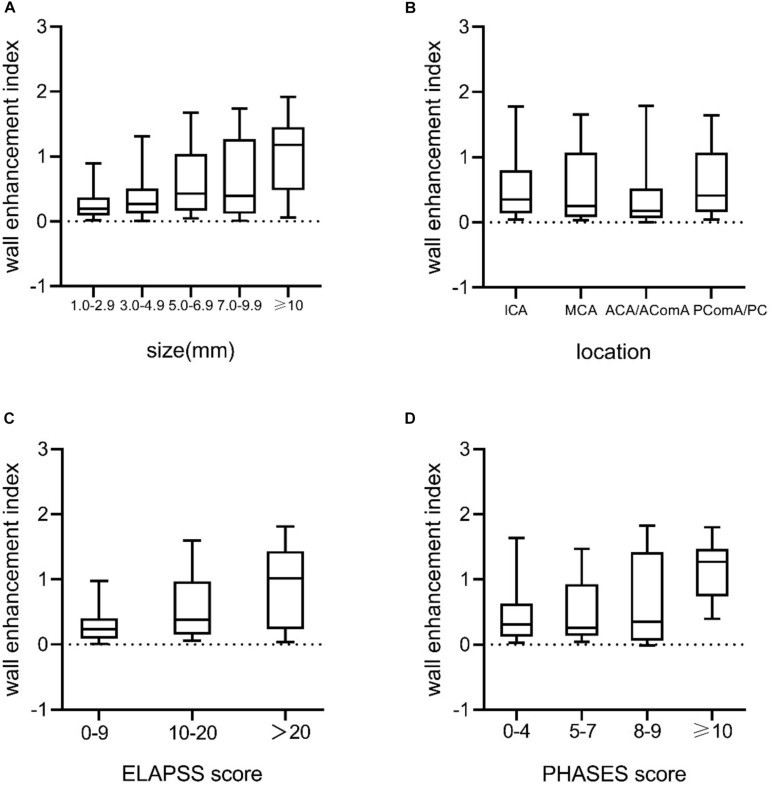
Box-and-whisker plots of medians (lines within boxes), interquartile ranges (upper and lower ends of boxes), and 5th and 95th percentiles (bottom and top lines) for WEI according to **(A)** aneurysm size (mm), **(B)** location, **(C)** ELAPSS score, and **(D)** PHASES score. WEI, wall enhancement index; ICA, internal carotid artery; MCA, middle cerebral artery; ACA, anterior cerebral artery; AComA, anterior communicating artery; PComA, posterior communicating artery; PC, posterior circulation.

Univariate analysis revealed that aneurysm size, non-ICA/MCA location, and irregular shape were significantly associated with AWE (*P* < 0.05). Multivariate logistic regression analysis revealed that aneurysm size (OR, 1.3; 95% CI, 1.2–1.4*; P* < 0.001), non-ICA/MCA location (OR, 1.9; 95% CI, 1.0–3.6*; P* = 0.045), and irregular shape (OR, 2.4; 95% CI 1.2–4.5; *P* = 0.009) were independently associated with AWE (Table 2). The proportions of UIAs with different enhancement patterns demonstrated significant differences in aneurysm size, location, ELAPSS score, and PHASES score (*P* < 0.001, *P* = 0.013, *P* < 0.001, and *P* < 0.001, respectively; Figure 4). The box-and-whisker plots showed the wall enhancement index in different groups according to aneurysm size, location, ELAPSS score and PHASES score (Figure 5). Larger areas and higher WEIs of AWE were positively associated with aneurysm size (*r* = 0.43 and 0.38, respectively; *P* < 0.001).

**TABLE 2:** Multivariate logistic analysis for risk factors independently related to aneurysm wall enhancement.

Variable	Odds ratio	95% confidence interval	*P*-value
Size	1.3	1.2–1.4	<0.001*
Location	1.9	1.0–3.6	0.045*
Irregular shape	2.4	1.2–4.5	0.009*
Early SAH history	3.6	0.9–15.3	0.077
Multiple aneurysms	0.9	0.5–1.8	0.811

SAH, subarachnoid hemorrhage, *P<0.05.

### Aneurysm Wall Enhancement Characteristics and Predicted Aneurysm Instability

**FIGURE 6:**
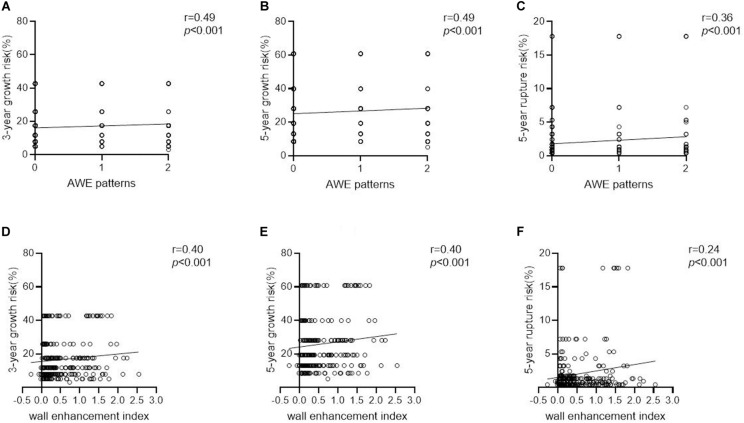
The correlations between AWE patterns/WEIs and aneurysm growth/rupture risk. **(A–C)** The AWE patterns and aneurysm growth/rupture risk. **(D–F)** The WEIs and aneurysm growth/rupture risk).

Aneurysms with AWE had a 1.9–3.3-fold higher estimated growth risk (3- and 5-year, 25.3 ± 13.0% and 38.0 ± 17.4%, respectively) than did aneurysms without AWE (12.8 ± 9.1% and 20.3 ± 13.0%, respectively; both *P* < 0.001). Similarly, aneurysms with AWE had a greater than threefold higher estimated 5-year rupture risk than did aneurysms without AWE (3.9 ± 5.2% vs. 1.2 ± 1.6%, *P* < 0.001). Larger areas and higher WEIs of AWE were positively correlated with the 3- and 5-year growth risk and 5-year rupture risk (*r* = 0.49 and 0.40, *r* = 0.49 and 0.40, and *r* = 0.36 and 0.24, respectively; all *P* < 0.001). The correlations between AWE patterns/WEIs and aneurysm growth/rupture risk are shown in Figures 6A–C: the AWE patterns and aneurysm growth/rupture risk; D F: the WEIs and aneurysm growth/rupture risk).

## Discussion

This study qualitatively and quantitatively assessed the degree of AWE. Black-blood MRI revealed that AWE patterns and WEIs were associated with risk factors related to rupture and growth and to clinical ELAPSS and PHASES scores. These results indicate that AWE is a non-invasive imaging marker that may provide information on aneurysm instability. However, these markers require validation in larger-scale longitudinal studies for predicting aneurysm growth and rupture. To the best of our knowledge, this is the largest study to date (*n* = 248) of asymptomatic UIAs that applies quantitative measurements of AWE. This is also the first study to correlate AWE with both ELAPSS and PHASES scores.

Individual prediction of aneurysm growth and rupture risk is critical for patient management. Risk and benefit must be balanced for surgical intervention or patient surveillance. Previous studies have demonstrated that AWE is associated with unstable aneurysms, which are defined as aneurysms with symptoms, growth, and rupture. Edjlali et al. (2014) and Fu et al. (2018) demonstrated that AWE was the only independent factor associated with aneurysm instability. The first longitudinal study (Vergouwen et al., 2019) of 65 aneurysms with 146 aneurysm-years of follow-up revealed that an outcome event (growth or rupture) was observed more frequently in aneurysms with enhancement during follow-up, which illustrated that AWE is associated with an increased risk of aneurysm instability. Omodaka et al. (2018) reported that the degree of circumferential enhanced aneurysm wall in evolving aneurysms was higher than that in stable aneurysms and lower than that in ruptured aneurysms. These findings suggest that AWE may contribute to the prediction of aneurysm instability.

AWE is a potential imaging biomarker that reflects wall inflammation. Inflammatory cell infiltration, cytokine secretion, neovascularization, and vasa vasorum formation reflect inflammatory responses in the aneurysm wall and may promote the degeneration and instability of the aneurysm wall, thus participating in aneurysm rupture (Hasan et al., 2012; Marbacher et al., 2014; Larsen et al., 2018). Quan et al. (2019) reported that AWE in unruptured aneurysms strongly indicated inflammatory infiltration of the UIA wall because it was significantly correlated with a high expression of inflammatory markers, including CD68 macrophage cells and anti-NLR Family Pyrin Domain Containing 3 (NLRP3). NLRP3 is an inflammasome that controls caspase-1 activity and pro-interleukin (IL)-1, leading to the development of atherosclerosis (Karasawa and Takahashi, 2017). Hu et al. (2016) reported that ruptured aneurysms contained abundant lymphocyte and macrophage infiltration. Further, high-resolution magnetic resonance imaging (HR-MRI) demonstrated enhancement in the corresponding area. These results indicate that AWE may be employed as an imaging biomarker for aneurysm wall inflammation and is valuable for assessing aneurysm instability.

In addition to inflammatory responses in the aneurysm wall, clinical and conventional characteristics—including regional population, age, hypertension, an earlier history of SAH, and aneurysm size, location, and shape—have contributed to aneurysm instability, including growth and rupture risk. The ELAPSS (Backes et al., 2017) and PHASES (Greving et al., 2014) scores are based on these characteristics. Determining the relationship between wall enhancement and these traditional risk factors enables the employment of AWE to predict the rupture risk of incidental UIAs. Lv et al. (2019) reported that aneurysm size and ACA/PComA/PC location were independently associated with AWE, and AWE was detected more frequently in UIAs with a higher estimated rupture risk. A larger aneurysm size (Murayama et al., 2016) in basilar tip, in the overall vertebrobasilar location, and in the posterior communicating artery location (UCAS Japan Investigators et al., 2012), as well as irregular shape (Pierot et al., 2020), have been reported to be related to ruptured aneurysmal status. This study complements past findings and further establishes the relationship between qualitative and quantitative vessel wall imaging findings, rupture-associated factors, and aneurysm instability, thereby confirming the utility of using AWE as a relevant biomarker in clinical practice.

One strength of our study was the use of objective and quantitative assessment of wall enhancement, which involves reproducible methods and may provide more information than simple qualitative grading. Previous studies (Nagahata et al., 2016; Zhu et al., 2018) classified AWE as different grades such as no, faint, or strong enhancement compared to choroid plexus or venous plexus, and pituitary infundibulum based on human observation. However, the methods of assessing AWE are partly subjective, and reference guidelines have not been standardized. Our study used a more precise indicator, namely, quantitative WEI calculations, to represent the degree of enhancement. Edjlali et al. (2018) reported that the WEI was significantly higher in ruptured aneurysms than in unruptured aneurysms and concluded that higher WEIs could reflect the rupture state. Roa et al. (2020) compared several quantification methods of UIAs enhancement and found the CRstalk (maximal aneurysm wall signal intensity/pituitary stalk signal intensity) was the most reliable objective method to differentiate >7 mm aneurysms. However, a level >7 mm is not a true standard of aneurysm stability, and the pituitary stalk is known to change signal dynamically depending on the time after contrast injection. Future studies are still needed to standardize the quantification of AWE. Our study demonstrated that larger areas and higher WEIs in asymptomatic UIAs were positively associated with larger size and increased aneurysm growth and rupture risk, which indicates that AWE may be a useful biomarker for clinical instability assessment.

### Limitations

Our study has several limitations. First, this was a single-center study, limiting the generalizability of the findings. Second, this was a cross-sectional study, and we did not have follow-up data for enrolled patients. Long-term follow-up to determine growth and rupture of aneurysms is needed to validate the AWE patterns and WEI. Thirdly, some of the aneurysms were imaged by 2D vessel wall imaging with 2mm slice thickness. The 2D imaging is limited in the evaluation of small aneurysms. However, only a few patients (30 of 174, 17.2%) had 2D scans, and among them only 10 patients had aneurysm size < 5mm (5.7% of the entire cohort). Given this small percentage, we believe the influence on our results is small. Fourthly, PHASES score had its own limitation that didn’t include morphological data, and the Chinese population as included in this study was not included in the PHASES studies. Therefore, our results should be interpreted with caution.

## Conclusion

We demonstrated that larger aneurysm size, non-internal carotid artery/middle cerebral artery location, and irregular shape were independently associated with AWE. Larger AWE areas and higher WEIs were associated with an increased risk of aneurysm growth and rupture based on ELAPSS and PHASES scores. These findings suggest that quantitative AWE metrics should be considered in future large-scale longitudinal studies to evaluate their value in aneurysm risk management.

## Data Availability Statement

The original contributions presented in the study are included in the article/supplementary material, further inquiries can be directed to the corresponding author/s.

## Ethics Statement

The studies involving human participants were reviewed and approved by ethics committee of the First Affiliated Hospital of Zhengzhou University. The patients/participants provided their written informed consent to participate in this study.

## Author Contributions

YZ and QF were responsible for patient recruitment, data acquisition, and/or analysis. JC, CR, SG, and CZ contributed to the study design. QF and CZ contributed to the imaging sequence development and optimization. YZ, QF, YW, and CZ contributed to the statistical analysis and drafting of the manuscript. All authors read and approved the final manuscript.

## Conflict of Interest

The authors declare that the research was conducted in the absence of any commercial or financial relationships that could be construed as a potential conflict of interest.
